# Unusal Cause of Crampy Abdominal Pain

**DOI:** 10.4103/1319-3767.65191

**Published:** 2010-07

**Authors:** Vipul D. Yagnik

**Affiliations:** Department of Surgery, Pramukhswami Medical College, Karamsad-388 325, Gujarat, India

A 40-year-old gentleman came to surgical OPD with chief complaint of crampy abdominal pain since last 12 days. He had complaint of occasional vomiting. Physical examination was not contributory. On per rectal examination, finger was stained with blood. His past surgical and medical history was not significant. His vitals were normal. After routine investigations, Barium enema was advised and it showed the findings revealed in Figure 1.

**Figure 1:**
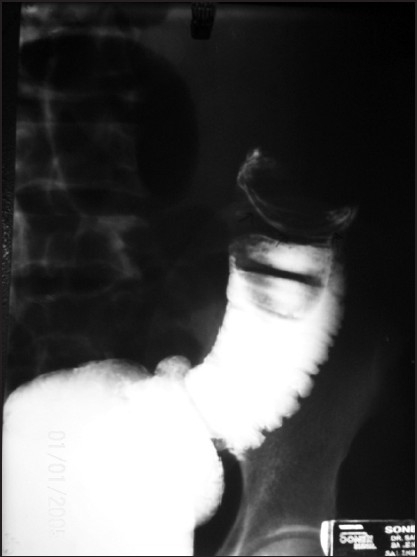
Barium enema.(Reproduced with permission from Fundamentals of Operative Surgery by Vipul Yagnik, Published by BI Publications, New Delhi)

## QUESTIONS

1. What is the diagnosis?
2. Which are the common lead points for Colocolic intussusceptions?
3. How to differentiate between non-lead point and lead point intussusception?
4. Enumerate the signs of intussusception on USG?

## ANSWERS

1. Barium enema revealed typical claw sign in left lumber region suggestive of colocolic intussusceptions.
2. Colocolic intussusception in the adults is almost always due to pre-existing disease like carcinoma or polyp.[1]
3. Abdominal CT scan with contrast helps in differentiating non-lead point and lead point intussusception.
4. Transverse section revealed ‘Doughnut sign and longitudinal section showed Bull’s eye and pseudo kidney sign.[2,3]
